# An Address to the Graduates of the Medical Department of the University of Buffalo, February 24th, 1869

**Published:** 1869-03

**Authors:** Thomas F. Rochester

**Affiliations:** Professor of the Principles and Practice of Medicine and Clinical Medicine


BUFFALO

### Medical and Surgical Journal.

VOL. VIII. MARCH, 1869. No. 8.

### Original Communications.

### HYGIENE.

ART. I.—*An Address to the Graduates of the Medical Department of the University of Buffalo, February 24th,* 1869. BY THOMAS F. ROCHESTER, M. D., *Professor of the Principles and Practice of Medicine and Clinical Medicine.*

'Tis a summer's morn in ancient classic Athens. The golden sun, rising refreshed from his purple couch, is gilding mountain and hill and temple-top and every lofty object, with that bright beaming effulgence which beautifies wherever it gleams, and gives life and warmth to whatever it touches. Westward from the fair islands of Ionia, greeting the god of day, comes a gentle and welcome breeze, nerve-bracing, health-giving, carrying in its breath, like a laugh, the joyous refrain of the thousands of feathered songsters who fill the air with notes of praiseful melody. Morning is just springing from the dark clasp of night, but the whole city is astir. Crowds in gay attire throng the avenues; children, their light locks sporting with the wind; young men and maidens with glistening eyes and blushing cheeks whispering the old-new tale; stalwart warriors plumed and armored; stately matrons grave and proud; old men and aged women marked by the stamp of time; the care-worn invalid; the halting cripple; philosophers, stoic and epicurean—and the *oi polloi*, the great nameless mass, all are there, and their course, on steed, in chariot and on foot, is toward the grand citadel—the lofty, far-famed Acropolis. 'Tis plainly a festal day. What shrine is to be honored? What deity to be invoked? There stands the lordly statue of Apollo—god of the fine arts—medicine, music, poetry and eloquence. Here the majestic Minerva, proud maiden goddess of wisdom and of war. Between and a little retired—oh! admirable juxtaposition—graceful, buoyant, modest, draped in a flowing robe, with hands outstretched and body poised on springing foot, is a charming female figure. Garlands hang upon and around her. Fresh flowers carpet every approach and float in the libations of generous wine that flow in streamlets past her feet. The pipe and the song are heard. The merry dance goes on, and ever and anon, the grand chorus, the constant attendant of the Grecian muse, sounds in rapturous strain the praises of Hygeia—Hygeia the daughter of Æsculapius—goddess of health.

Nearly three thousand years have come and gone since the gay pageant just described was enacted. The Prince of Peace came, and the ideal creations of by-gone ages were scattered like the myths they were. The "unknown God," revealed and proclaimed in the same Athens, on the Hill of Mars, by the great apostle of Tarsus, is now the acknowledged and reverenced Great Father and Creator of the world, and all that therein is. Let us look again at south-eastern Europe—the scene only removed from that first depicted, by the dark waters of the stormy Euxine. 'Tis Anno Domini 1854; Papal France and Protestant England have rushed to the succor of the infidel Turk, menaced by the strong arm of their christian brother—the feared, dreaded, and envied Muscovite. Fierce is the struggle—terrible the carnage, but great as is the mortality from the enginery of war, greater is the loss from cold and wet and heat—from improper and insufficient food, from deadly pestilence generated and propagated by an utter disregard of all sanitary measures, and—oh! worse than all—by the fearful condition of the immense but crowded, filthy and unventilated military hospitals. The charnel houses of Scutari and Odessa, were justly more dreaded than the fierce metallic hurtle that poured without cessation from the molten throats of beleaguered Sebastopol.

The battle of Balaklava has just been fought, and the parched earth is moistened deep and strong by the red torrent that flowed so full on the dread field of bloody Inkerman. Where shall the sick and the wounded find succor? "Oh, leave me in God's fresh air," said one who had already tasted, and escaped with life, the horrors of the hospital. Poor sufferer! he knew not the transformation a few short weeks had effected. A band of women, led by one who had for years made sorrow and pain and sickness a study of love, had taken upon themselves the devoted task of cleansing, purifying and regulating these plague spots of filth, neglect and incompetence. Neatness, order, promptness, obedience, fresh air, sunlight, suitable and sufficient food, reading matter, writing material, friends, advisers, sympathisers, substituted for the indescribable condition of preëxisting months, proved how well they had succeeded. Of her who presided over these true sisters of mercy, the *Times* correspondent wrote: "She is a ministering angel in these hospitals, and as her slender form glides quietly along each corridor, every poor fellow's face softens with gratitude at the sight of her." And again: "With the heart of a true woman, and the manners of a lady, accomplished and refined beyond most of her sex, she combines a surprising calmness of judgment and promptitude and decision of character." Listen, too, to this homely, yet most poetic testimony of a wounded soldier: "She would speak to one and to another, and nod and smile to as many more; but she couldn't do it to all, you know; we lay there by hundreds, but we could kiss her shadow as it fell, and lay our heads on the pillow again, content." Another soldier said: "Before she came, there was such cursing and swearing; and after that, it was as holy as a church." 'Tis the living Hygeia of the nineteenth century. *Dea patuit*, and her shrine, the hospital ward. Ah, noble woman! how many canonized saints have equaled thee in devotion? Hail! Florence Nightingale, sister of Anglo Saxon blood, exemplar and prototype of Margaret Breckenridge, and of other hundred heroines, no less patriotic and devoted.

The pictures are before you, gentlemen—Pagan poesy—Christian utility. The former has many attractions, and, to the mere theorist, presents many affinities. Golden dreams of subtle life-essence, controlled by impalpable and imponderable agents, are pleasant to revel in. Mysticism and credulity find propagandists and victims in the imaginary potencies of inappreciable attenuations and immeasurable dilutions, but the pure scientist treads the hard path of fact, tried and tested by the searching analysis of reason. All mankind seek that inestimable blessing—health. The aesthetic Greek, refined by culture, but unenlightened by revelation, sought it through the benignant influence of a female deity—herself the pure creation of mind—of mind, that divine attribute of man, which, feeling its need, creates what it cannot find, and makes almost true, the ideal of the unattained material. With the same end in view, the mortal of the nineteenth century, better instructed in the mystery of his being, strives to accomplish his purpose, by employing, uncontaminated, what by usage he calls the gifts of nature, meaning thereby the concordant creations of God.

You, gentlemen, have chosen as a profession—medicine, a word derived from the Latin *Medicare*, to heal—but you have read and listened and thought in vain, if you have not learned that it has a broader significance, a wider scope. Confucius was right when he asserted that it was the province of the medical man to prevent rather than to cure disease. You have studied the anatomy and physiology of man; you have seen and made yourselves acquainted with the disorders with which he is afflicted; you have learned how drugs and appliances are employed to control the latter, and it is to be hoped that you have also learned that much of what we call disease, or piously ascribe to "visitations of Providence," arises from ignorance or from willful neglect of known, baneful agencies. To prevent and counteract these and their effects, will constitute no small part of your labors. Fame, success and philanthropy all urge upon you, unwearied devotion to that most essential part of your profession, which we call, often in unconscious homage to the Grecian Diva—Hygiene.

It needs not the battle strife and its sequences of woe; it needs not the pestilence and its horrors, to afford a field for work. It is here at our doors, in our midst, and oh! shame to human intellect and witness of human frailty—the field is often widest where we boast of refinement, progress and reform. Fresh air—sunshine—personal and general cleanliness, and good food properly prepared, are the chief promoters of healthfulness and vigor. These are within the reach of all save the very poor. Let us see whether we use them or not. We are proud of our public schools; education is free to all, but it is not in every instance the blessing that it seems; it is acquired at too great an individual and general risk. On the proudest avenue of this thriving city is a three storied brick building, Public School No. 10. The first floor is devoted to the primary department. The room is heated with stoves, the ceiling is low, the light is but moderate; there is no provision for ventilation. The seats are short and narrow and close together. Eighteen months ago a meeting of the residents of the district was convened to consider the propriety of repairing the old, or of building a new structure. The principal of the school, in reply to inquiries, stated that the room was always full, that three children had to sit where there was only room for two, that they were packed so thickly that it made it impossible for the children all to rise upon their feet at once; that there was no place to hang up their outer garments, even if they were wet, and that when school was dismissed, if a boy should drop his cap, he could not stop to pick it up so great was the "rush and crush." The tax-payers—a majority of them—buttoned up their pockets and voted to repair; one of them, a wealthy professional man, remarking, "that it was a much better school house than the one he had studied in." Not so, sir! The little, wooden, country school, with its rattling windows, its gaping cracks, and its roaring log fire in the broad chimney, was infinitely superior.

On the ninth of February, 1869, a fortnight ago, the school committee of the common council with the superintendent of schools, made a tour of inspection. I make a few extracts from the report of the same, published in a morning paper of the 10th instant:

"No. 7. The primary department was found to be running over with little children, who have hardly room to breathe and stretch out their little arms."

"No. 11. It is a perfect hive of children; in one room there are two hundred in daily attendance."

"No. 31. The primary department has three hundred and forty scholars, but was calculated only to hold one hundred and eighty. They sit everywhere."

"No. 15. The primary department contained three hundred and twenty scholars yesterday."

From eight to twelve hundred cubic feet of air, with good ventilation, is the amount of space that is required to be alloted to each individual in the United States military hospitals. In British India each jail prisoner has by legal enactment six hundred and forty-eight cubic feet of air. In public school No. 15, each poor child has but fifty-six cubic feet of air. All the public schools are not like these, but that there should be one such, is a burning shame. It is the primary department, this place where the child of five years old and upward, the little darling that yet needs a mother's watchful eye and care, is subjected to such infamous trial and exposure. No wonder that scarlet fever, diphtheria, typhus and typhoid fever, and blood poisons of every description are always more or less prevalent. A large proportion of the cases of these dread disorders are generated in, and propagated by our public schools, and the sordid man who votes against a tax for health and education, cannot guard his own spacious and luxurious abode against the malady his avarice has helped to originate. But acute diseases are not the only result of this criminal crowding. Tuberculosis and brain affections developed at various periods, may be traced but too often to the same source. Better for society, and better for themselves would it be, that these infants were not educated at all, than at such risks. Is it strange that the teacher is wan and pale and weary? Even the developed adult brain and frame are not proof against the trial.

In vain have superintendent and teacher protested for years against the evil. It is for you, my hearers, as parents and good citizens, to stop this slaughter of the innocents.

Now let us pass from these school rooms—these pest rooms rather—to our dwelling houses, and first to those of the wealthier class, almost any one on the same avenue *graced* by "No. 10." The rooms are large, the ceilings are high, comforts and luxuries abound. The apartments are not crowded, at least with people; but the indoor air seems close and rather warm. The elegant thermometer, resting on the fireless mantel marks a temperature of 75 degrees, generally higher. You step near the register and shrink from inhaling the hot, dry blast which strikes you. There are double sashes to the windows, and heavy curtains within. The outer doors are double also, the throat of the chimney is closed—everything to keep out the fresh air and to keep in the hot. The elegant furniture is dried and cracked. The doors are warped and shrunken. Light chairs fall apart, and the cabinet maker is blamed. Dear madam, they are only doubly kiln-dried! The singing bird on his gilded perch, rarely utters a note; poor fellow! his cage hangs two feet higher than your head, and his throat is parched and his tongue shrunken, for it's very warm up there! There are no plants and fragrant flowers blooming, but their "counterfeit presentment" in ornate vase is seen. This is the atmosphere in which our wives, our daughters, our little children, *exist* for at least half of the year—an atmosphere in which a hot-house plant cannot live. The men and boys can rush out doors and get away. Not only is this *dried* air breathed in the "living room," but it also invades the bed chamber, and often, both by day and by night, is it tainted by the foul fumes of escaping coal gas. What is the effect of this elevated temperature, and dried and deoxydized air? The mother has headache and is cold, rather than hot. The children have big heads, pale faces and little necks; and when an outer door is left ajar, all of them shrink from the invigorating breath of heaven, and seek the torrid, belching blast.

The hot-air furnace is doing much to enervate us as a people. We have not time this evening to further enumerate the present and prospective ills that result from it, but common sense asks: "Why is it used at all?" The dealer, and very likely the good housewife, will reply, "For comfort—for neatness—for economy." The first in every sense we deny; the second we admit and count it very small gain; the last we most emphatically repudiate, save that it sustains the axiom of Malthus, "that it is cheaper to bury than to feed." People who live in houses not overheated by furnaces and by similar *base* burning contrivances, are as a rule healthier and warmer than those who "cannot exist without them." But a grievous error is too often made in the selection of the sleeping apartment of persons of moderate means. From the fact that it "saves steps," a first floor bedroom is generally regarded as a desideratum, and a space of eight feet by twelve is often the dormitory, not of one person, but of several. In one corner there may be a window—usually darkened by closed shutters, and when, at rare intervals the sash is raised, neither the prospect nor the odor from without is agreeable. But too often, even at mid-day, does the medical man require a light to see his patient, in these closets, (closet from *close.*) The dread of running up and down stairs does not exist in Europe—the *troisieme* and the *quatrieme* are often selected from choice—and there is no good reason why the back of the American woman should be weaker than that of her Gallic or Teutonic sister. In our hotels, and especially in those immense caravanseras styled "first class," there is a fine parlor to which one or two bedrooms are attached, having no direct communication with the external air. There is a head-light over the door and a small sash, rarely opened, looking into a dim, dusty and noisy hall. It is difficult to over-estimate the risk to which these unventilated recesses expose the unwary traveler, necessarily ignorant of the condition of the preceding occupant. Clean sheets will not avert the poison of nascent small-pox or typhus. But some of you will naturally inquire how is it with the dwellings of the poor? Ah! poverty! with insufficient food and scanty raiment and vice, (tempter and beguiler of ignorance and destitution as well as of luxury and pride) with how many ills beset—how often do you reject the only boon that is within your reach—fresh air! We pass over the nameless horrors of the tenement house—foul offspring of pitiless avarice—and look at the hut and cabin. The rooms are one or two. The location from its insalubrity, the cheapest. Without, pigs and poultry—those domestic scavengers, themselves as noisome and filthy as the garbage they consume. Within, the mingled fumes of tobacco, whisky, cabbage, onions, exhalations from unwashed, perspiring human beings, and a mist like a cloud curtain, the product of the begrimed wet floor, and of the dripping clothes hung around the cooking stove, prolific of its own shocking aroma, even the dogs, the ducks, and the pigs, who often share this shelter on terms of equality—with men and women and countless children, even these, contract disease from the contaminated air of the hovel.

From the contemplation of such repulsive scenes, we gladly turn to our public halls and churches. They are usually spacious and often elegant, but they are almost invariably lamentably deficient in ventilation. The congregation are drowsy, and pronounce the clergyman "stupid;" he, poor man, who gives them brain-thought and work, not for the pittance upon which he manages to subsist, but from earnest interest in their future welfare, is disheartened by their dullness and indifference. Pardon, good pastor and people, you are both in error. The fault is neither mental nor psychical—it is physical. By the breath and the skin the animal body gets rid of much of its used up material in the shape of carbonic acid. This noxious agent when inhaled or absorbed, produces stupor. Its density is greater than that of pure air. A hole in the roof, or aloft at the side, allows hot air, which rises, to escape, but the carbonic acid remains below. A great deal of time and thought and money have been expended uselessly on ventilation; complicated machinery has been essayed, and has generally failed. There is one church* in this city that *is* completely ventilated, and yet it is always warm and comfortable, and without thorough draft. The clergyman is not "stupid," nor are the people "dull." It is a very slight matter. The ventilation is from below. At one end a large chimney with a wide expanse, opens on the floor level—its flue, straight and broad, rises above the roof. A second flue in the same stack carries off the hot smoke and gas of the draught-pipe of the furnace which warms the house. This latter flue thus heated, warms the air in the former, and as the hot air in it rises, the broad chimney draws in and carries off the air charged with carbonic acid. If sufficient fresh air does not enter through the doors and window frames, openings in the walls are uncovered for that purpose. This easy and admirable method of ventilation, may be applied to any large public room, and does away with the objection of the most practicable way of warming it, that is, by furnace heat.

In this connection, brief allusion may be made to the excessive heating and inadequate ventilation of our railway cars. As a rule, too many people are crowded into each carriage, the fires are too hot and the ventilators are kept closed. More "colds are taken" from bad air and from excessive heating than from insufficient warmth. Sooner or later we shall go back to the compartment system (elegantly illustrated by the drawing-room cars) and with extra wrappings get warmth enough, and avoid the worst part—the burning part—of the terrific disasters of Angola and Carr's Rock.

---

* The Lafayette Street Church.

It would enable us also to dispense with that questionable luxury, the sleeping car.

"*C'est ne que un sot qui batte la mode*"—fools only fight fashion, is a French proverb. As we do not wish to be classed in that category, we are glad to be able to testify that fashion and common sense are of much more accord than formerly as regards the out door equipment of females and children. The thin slipper is supplanted by the stout walking shoe. The train, graceful and appropriate in the drawing room, no longer sweeps the street. The chignon, which a South Sea Islander might envy, is not really as bad as it looks, and the jaunty hat, hanging over the brow, protects the eyes and the forehead, preventing headache and facial neuralgia. The bare necks and limbs of children are no longer paraded in the open air, to the great peril and discomfort of the little ones. But fashion has many sins laid to its charge—and not without reason. There is certainly fashion in medicine. Witness this and that absurd system, which every now and then seizes the sick, or imaginary sick public; fashion in theology—witness, ritualism; fashion in morals—witness, the atrocious soul and body imperilment by the avoidance of the sacred duties of legitimate maternity, by practices both indelicate and immoral. This is called an American fashion; it is certainly a fashion, an immorality, a vice, but it is not an original American fashion. It is derived from that people who are Fashion's chief votaries—who dethroned the God of the Bible, and made adoration of a day to a painted creature—miscalled reason. Medical men were the first to sound the alarm, both in its moral and physical aspects. Philosophers and statisticians re-echo it, in its national and generic relations. The religious and the secular press alike expose and denounce it, and the pulpit implores, persuades and fulminates against it. This usage is not limited to any people, nor is it avoided, as has been stated, by those of any creed. (Medical men know this.) It is one of the banes of civilization. That it finds more congenial soil in our country than elsewhere, cannot, alas! be denied. Poverty and inconvenience are urged in extenuation. These do not excuse infractions of human law, much less of divine; but even the excuse is false; the real culprits are fashion, frivolity, laziness, selfishness, and O tempora! O mores! national demoraliztion. Let no one charge that our language is too plain. When persuasion and argument fail in private, the public heart must be stirred. If we would exist as a nation, this generic suicide must be stopped. Let us not follow, but be warned by the example of France, whose population is steadily decreasing, not from migration, war or pestilence, but from unnatural causes, the death rate exceeding the birth rate by at least 50,000 per annum.

Your attention is now invited to a subject of more importance than, from its title, you would perhaps imagine, to wit: *our food.* Upon it depend not only bone and muscle, but also intellect and brain. Man is an omniverous animal, and derives his complete strength and development from the properly combined nutrients of the animal and vegetable kingdoms. We have them all; not only in sufficiency, but in profusion. How do we use them? We are alive to the dangers of trichinatous pork, (from the investigations of medical men) and we are painfully cognizant of the horrors of diseased and bruised beef. Against these we hope to be protected by appropriate legislative action, but the *vis vitæ* that is thrown away by improper cookery, and by the repeated siftings of our cereals, few but men of thought and science suspect. The juices of both meats and vegetables are mostly wasted by maceration (in boiling) or by the charring (in roasting) to which they are subjected, and thus the nitrates and phosphates—the intellect and nerve-feeders—are lost. Except fruits, all food should be cooked, and thoroughly so; but in such manner that all its ingredients should be preserved. Upon this depend not only flavor and digestibility, but also nutritive properties. The selection of food is not less important than its preparation. Pork and beans are very well for the plowman, but very *unwell* for the man of sedentary habits, or of intellectual avocation. For the latter a fish diet would be far preferable, as it supplies the phosphorous consumed by energy of brain. A recent and forcible, if not elegant writer, says:

"Our Puritan forefathers, who lived on beans, peas, unbolted grains, and the meats, vegetables and fruits, as they came from their fields and gardens, cooked in the simplest manner, best calculated to develop their natural flavor and prepare them for digestion, were not troubled with flatulence, colic and indigestion, and our foremothers were not the pale-faced, flabby-muscled, toothless, chlorotic, consumptive and sentimental race, as are their degenerate daughters of the present generation. Even our farmers and their wives and daughters have become terribly degenerated. Instead of the robust and healthy men, and the full-chested, healthy, rosy-cheeked, beautiful women of former generations, we see a people almost as feeble and sickly as the city people. And the reason is apparent. The outer crust of the wheat, and the buttermilk which contain the nitrogen, phosphorus and iron on which strength and energy, mental and physical, and beauty of complexion depend, is given to the cattle and pigs, while they take themselves, instead, the butter, fine flour and sugar, which contain only the heating and disease-producing carbonates. The robust Irishmen and Scotchmen, also, who come here with strong, energetic muscles and sound teeth, from their oat-meal, wheat and barley cakes, with their potatoes, buttermilk and cheese, soon fall into our starch and grease-eating habits, and become, or at least their children become, as pale, puny and toothless, as pure-blooded Yankees."

Bread is called "the staff of life." When this name is given to it, as now prepared, is it not by poetic license? Good housekeeper, your white loaves and rolls, made from the "snow-flake" brand, are almost as evanescent as that from which the name is derived. A grand mistake, and made too often by physicians as well as others, is to think that articles which are simply fattening, are also strengthening. The feeding of the race-horse and the roadster is not conducted on this principle. White flour, starch, butter, oil, fats, sugar and alcohol, are carbonaceous material. It has its place in the animal economy, but it does not make good brawn.

Not only do we throw away much that is good in our grain products, but we also impair that of them which we keep, by conditions and combinations that are hurtful. Every dietist knows that hot bread is more or less indigestible; but its use might be allowed but for the detestable saleratus, or acido gypsum compounds, disguised as "baking powders," by which it is tainted, and its consumers are *tinted.* Canned fruits and vegetables are most of them latent poisons. There is more or less lead in tin, and a good deal of lead in the solder with which the cans are joined and closed, and large, loose lumps of solder are often left within. Vegetable acids act upon this lead, and we take in solution in our delicacies, what we try to avoid in our drinking water. In many a small can of tomatoes or peaches, there is more lead in solution than in a tun of hydrant water. Even the glass jars which are used for the same purpose, are not entirely innocuous, as some of them also contain lead. But apart from this most tangible objection; to mankind were given "the fruits of the earth *in their season*," and that which is healthful and appropriate in warm summer is quite the reverse in cold winter. The adulteration of food is carried to an extent hard to credit, and the same thing is true as respects drugs, which of all things should be pure; but upon these topics we have no time to dwell this evening. For the same reason we can but allude to the location and surroundings of dwellings—to the question of drainage, paving, and pure water supply—to the influence of trade or occupation upon the physical and mental organism, and to various kindred subjects. As medical men you must think and act upon all these matters. From your professional position you must study them, and you will find their consideration no less important than the investigation of disease, and the application of therapeutic agents.

Gentlemen! You are about to embark in a career which has many trials, but also many rewards. In proportion to your disinterested and earnest efforts for the welfare of your fellows, will be your success. But that you may promote the health and happiness of your neighbors, it is highly necessary that you should take care of yourselves. It is all important that you should possess in its truest sense, a home; you must have your own Lares and Penates, and above all your own domestic Hygeia—a good and faithful wife—for she is "a crown to her husband" and "her price is far above rubies."

